# Antiandrogenic treatment of obsessive compulsive neurosis: A case review

**DOI:** 10.1192/j.eurpsy.2023.1961

**Published:** 2023-07-19

**Authors:** L. Huerga García, I. Careno Baez, G. Oropeza Hernández, A. Marcos Rodrigo, C. Delgado Torres, G. Garriga Rocío, P. Gómez Pérez

**Affiliations:** Psychiatry, Hospital Universitario Nuestra Señora de la Candelaria, Santa Cruz de Tenerife, Spain

## Abstract

**Introduction:**

Obsessive-compulsive disorder (OCD) is a mental disorder in which patients who suffer from it have repetitive and undesirable thoughts, feelings, ideas, sensations (obsessions) and behaviors that drive them to do something over and over again (compulsions).

Often the person tries to get rid of the obsessive thoughts through compulsions, but this only provides short-term relief. Not carrying out the obsessive rituals can cause enormous anxiety and suffering.

**Objectives:**

To describe a 23-year-old male patient, who suffers from anxiety and mood symptoms, reacts to ego-dystonic obsessive ideas and sexual content, of months of evolution, and who manages to calm down through compulsive masturbation or watching sexual videos on the internet. All this clinic negatively interferes with their quality of life, asking the patient for medical help to calm these ideas.

**Methods:**

We carried out a review in Pubmed with the terms Antiandrogens and TOC, in order to make a better description of the clinical case.

**Results:**

After several treatment attempts (Sertraline, Paroxetine, Clomipramine, Clomipramine + SSRI), reaching maximum doses according to clinical guidelines, and with poor therapeutic response, it was decided to discuss the case with the endocrinology department of our hospital, deciding to start treatment with antiandrogens, in order to alleviate the persistent intrusive ideas of a sexual nature. The administration of antiandrogens in men can cause a decrease or increase in the development or involution of secondary sexual characteristics in men, reducing the activity or function of accessory sexual organs, and hyposexuality, with decreased sexual desire or libido.

After several weeks, there was improvement in the obsessive symptoms with a decrease in compulsive rituals. However, after the 3rd mo, some symptoms reappeared, but not with the same severity and intensity as before treatment. In addition, we cannot ignore the adverse effects that have occurred, such as involution of secondary sexual characteristics. However, and taking into account the negative repercussion that this clinic had on the patient’s quality of life, the benefit obtained exceeded the risk, having noted clear improvement with this therapy, and maintaining evolutionary controls by both psychiatry and endocrinology.

**Conclusions:**

Patients suffering from obsessive-compulsive disorder can be effectively treated with anti-androgenic pharmacological agents with various modes of action. The most effective group of such agents is the long-acting analogues of the gonadotropin-releasing hormone. The objective of this review is to elucidate the possibility of using such powerful anti-androgenic agents in the treatment of obsessive-compulsive disorder.

**Disclosure of Interest:**

None Declared

